# Comparison of Menstrual Bleeding and Dysmenorrhea Following Immediate Postpartum Insertion of Copper-T 380A and Cu 375 Intrauterine Contraceptive Devices: A Prospective Observational Study

**DOI:** 10.7759/cureus.106715

**Published:** 2026-04-09

**Authors:** Deepti Dahiya, Chesta Garg, Aaria More, Reetu Hooda, Nisha Malik, Krishna Dahiya, Akta Bajaj

**Affiliations:** 1 Obstetrics and Gynaecology, Pandit Bhagwat Dayal Sharma Post Graduate Institute of Medical Sciences, Rohtak, IND; 2 Obstetrics and Gynaecology, Sikkim Manipal Institute of Medical Sciences, Gangtok, IND; 3 Obstetrics and Gynaecology, Darling Downs Hospital and Health Service, Toowomba, AUS; 4 Obstetrics and Gynaecology, Ujala Cygnus Group of Hospitals, New Delhi, IND

**Keywords:** cu 375, cu-t 380a, dysmenorrhea, long-acting reversible contraception (larc), menstrual blood loss, postpartum intrauterine contraceptive device (ppiucd)

## Abstract

Background

Immediate postpartum intrauterine contraceptive device (PPIUCD) insertion is an effective method of long-acting reversible contraception. Differences in copper surface area and device design between Copper-T (Cu-T) 380A and Cu 375 may influence bleeding and pain profiles. Data in the immediate postpartum setting remains limited.

Methods

This prospective observational study compared postpartum women who received Cu-T 380A (n=150) with Cu 375 (n=150) as PPIUCD following vaginal or cesarean delivery. Participants were followed at six weeks and six months. The primary outcome was menstrual blood loss assessed using the Pictorial Blood Assessment Chart (PBAC) and dysmenorrhea assessed using the Visual Analog Scale (VAS). Secondary outcomes included expulsion, abnormal vaginal discharge, unintended pregnancy, removal, and satisfaction.

Results

Baseline characteristics were comparable between groups. At six weeks, PBAC and dysmenorrhea scores did not differ significantly. At six months, Cu-T 380A users demonstrated higher PBAC and dysmenorrhea scores compared with Cu 375 users (p < 0.001). The absolute difference in PBAC scores was modest but statistically significant, and median values in both groups remained below thresholds typically associated with heavy menstrual bleeding. Safety outcomes, expulsion rates, and satisfaction were similar between groups.

Conclusion

The present study highlights that although both the PPIUCD options Cu-T 380A and Cu 375 have good patient acceptability and high satisfaction scores, Cu-375 as a PPIUCD causes significantly less menstrual bleeding, as well as less severity and duration of dysmenorrhea, when compared to Cu-T 380A. Since complications at follow-up are a major determinant in PPIUCD acceptance, Cu-375 is a lucrative option for women opting for reversible contraception after delivery.

## Introduction

Postpartum contraception is a critical component of maternal and child health, enabling optimal birth spacing and reducing unintended pregnancies. According to the 2019-21 National Family Health Survey report, the unmet need for contraception in India is 9.4% [1]. Immediate postpartum intrauterine contraceptive device (PPIUCD) insertion provides effective, long-acting, and reversible contraception before hospital discharge. It protects against unintended and short-interval pregnancies, thereby reducing the risk of adverse perinatal outcomes and their associated economic burden [2]. Despite proven safety and efficacy, the long-acting reversible contraceptive (LARC) methods are still underutilized [3]. The advantages of using PPIUCD for both users and service providers are well established [4]; however, concerns regarding bleeding patterns and dysmenorrhea remain important determinants of continuation and satisfaction among copper intrauterine device (IUD) users.

Long-acting reversible contraceptives, particularly IUDs, are highly effective, safe, and well-suited for immediate postpartum insertion, providing both convenience and sustained contraceptive coverage. Copper (Cu)-bearing IUDs differ in copper surface area and structural design. The Cu-T 380A contains a larger exposed copper surface area compared with the Cu 375, which incorporates copper along a flexible multiload frame. Increased copper surface area has been associated with enhanced local endometrial inflammatory response and prostaglandin-mediated vascular changes, which may influence menstrual blood loss and pain perception [5]. Structural differences in transverse arm design may further affect endometrial contact and uterine adaptation during postpartum involution. However, variations in bleeding and pain profiles between different copper IUDs may impact patient satisfaction and continuation rates, underscoring the need for evidence-based guidance to support individualized contraceptive counseling.

Although several studies have compared Cu-T 380A and Cu 375 in interval insertion settings, evidence specific to immediate postpartum insertion remains limited and inconsistent. The postpartum uterus differs physiologically from the interval uterus in terms of size, vascular remodeling, endometrial regeneration, and involution dynamics, which may alter device-related bleeding patterns. Additionally, many prior studies relied on subjective bleeding assessment rather than standardized quantitative tools. In view of this gap, the present prospective comparative study aimed to evaluate menstrual blood loss using the Pictorial Blood Assessment Chart (PBAC) [6], dysmenorrhea, safety outcomes, and patient satisfaction over six months among women receiving either Cu-T 380A or Cu 375 as an immediate postpartum intrauterine contraceptive method.

## Materials and methods

Study design and setting

This prospective observational comparative study was conducted in the Department of Obstetrics and Gynecology at Pandit Bhagwat Dayal Sharma Post Graduate Institute of Medical Sciences, a tertiary-care teaching hospital in Rohtak, India, from February 2020 to March 2021. The study compared menstrual bleeding patterns, dysmenorrhea, and safety outcomes following immediate postpartum insertion of two Cu IUDs: Cu-T 380A and Cu 375. Ethical approval for the study was obtained from the Institutional Biomedical Research Ethics Committee of Pandit Bhagwat Dayal Sharma University of Health Sciences (approval number BREC/19/Th/Gynae-11 dated 23/12/2019). Written informed consent was obtained from all participants prior to enrolment in the study. 

Participant selection

Postpartum women aged 18-40 years delivering at the study center were screened for eligibility. Women were eligible if they had a gestational age of ≥34 weeks and consented to immediate PPIUCD insertion following vaginal or cesarean delivery.

Exclusion criteria included hemoglobin <8 g/dL, postpartum hemorrhage, prolonged rupture of membranes (>18 hours), obstructed labor, unresolved puerperal sepsis, known uterine anomalies, active genital tract infection, or known hypersensitivity to copper.

Eligible women were counselled antenatally or during early labor regarding postpartum contraception and the available IUD options. Written informed consent was obtained from all women participating in the study.

Insertion technique

In women undergoing vaginal delivery, the IUD was inserted within 10 minutes of placental expulsion using long placental forceps, ensuring placement at the uterine fundus under aseptic precautions.

During caesarean deliveries, the IUD was placed at the uterine fundus through the uterine incision using ring forceps. Strings were left within the uterine cavity. The uterine incision was closed in two layers, taking care not to entrap the strings.

All insertions were performed by trained obstetricians experienced in postpartum IUD placement.

Follow-up protocol

Participants were scheduled for follow-up visits at six weeks and six months postpartum.

At each visit, a detailed history was obtained regarding menstrual pattern, pain, abnormal vaginal discharge, and any adverse symptoms. Clinical examination was performed to assess string visibility and signs of infection. Ultrasonography was performed when the strings were not visible or when expulsion was suspected.

Participants were advised to report earlier if they experienced excessive bleeding, severe pain, or suspected expulsion.

Outcome measures

Primary Outcome

The primary outcome was the impact on the menstrual cycle in terms of menstrual blood loss at six weeks and six months postpartum, which was assessed using the PBAC score described by Higham et al. [6]. The tool assigns graded scores based on the degree of saturation of sanitary products and accounts for clots or flooding. Scores were recorded daily and aggregated monthly. The PBAC has a reported sensitivity of 86% and diagnostic accuracy of 89% [6]. Dysmenorrhea was assessed using a 10-point Visual Analog Scale (VAS) [7].

Secondary Outcomes

Secondary outcomes included the following: expulsion (partial or complete), abnormal vaginal discharge, unintended pregnancy, and device removal. Patient satisfaction was assessed using a five-point Likert scale (very satisfied to very dissatisfied). Heavy menstrual bleeding was interpreted in the context of PBAC scoring, with values ≥100 generally considered indicative of heavy menstrual bleeding in clinical practice.

Sample size calculation

The sample size was calculated to detect a minimum clinically relevant difference of 7 points in PBAC score between the two groups, assuming a standard deviation of 10 points, with 80% power and a two-sided alpha level of 0.05. A minimum of 126 participants per group was required. To account for potential loss to follow-up, 150 participants were enrolled in each group, resulting in a total sample size of 300 women.

Statistical analysis

Data were entered into Microsoft Excel (Microsoft Corp., Redmond, WA, USA) and analyzed using IBM SPSS Statistics software, version 21 (IBM Corp., Armonk, NY, USA).

Continuous variables were assessed for normality of distribution. Variables demonstrating approximate normal distribution were expressed as mean ± standard deviation, whereas non-normally distributed variables were summarized as median and interquartile ranges (IQR).

Differences in PBAC and dysmenorrhea scores between groups were analyzed using the Mann-Whitney U test. Categorical variables were expressed as frequencies and percentages and compared using the chi-square test or Fisher’s exact test, as appropriate based on expected cell counts. All statistical tests were two-tailed. A p-value <0.05 was considered statistically significant.

## Results

Participant characteristics

A total of 300 postpartum women were included in the study, with 150 women receiving Cu-T 380A and 150 receiving Cu 375 as immediate PPIUCDs. The two groups were comparable with respect to baseline demographic and obstetric characteristics. There were no statistically significant differences between the groups in terms of age distribution, parity, history of abortion, body mass index, literacy status, rural residence, duration of rupture of membranes, or route of IUD insertion (Table 1). These findings indicate that the study groups were well-balanced at baseline.

**Table 1:** Baseline demographic and clinical characteristics of study participants Data are presented as numbers and percentages unless otherwise specified; continuous variables are expressed as mean + standard deviation; *p<0.05 is considered statistically significant. Cu-T 380A: Copper-T 380A

Characteristic	Cu-T 380A (n=150)	Cu 375 (n=150)	p-value *
Age group (years)			
18–25	81 (54.0%)	77 (51.3%)	1.000
26–30	61 (40.7%)	66 (44.0%)	
31–35	8 (5.3%)	7 (4.6%)	
Parity P1	57 (38.0%)	61 (40.7%)	0.891
Parity P2	57 (38.0%)	54 (36.0%)	
Parity ≥P3	36 (24.0%)	35 (23.3%)	
Abortions A0	92 (61.3%)	84 (56.0%)	0.621
Abortions A1	40 (26.7%)	47 (31.3%)	
Abortions ≥A2	18 (12.0%)	19 (12.7%)	
BMI (kg/m²), mean ± SD	23.7 ± 2.8	24.8 ± 3.1	0.452
Illiterate	34 (22.7%)	30 (20.0%)	0.949
Rural residence	101 (67.3%)	98 (65.3%)	1
Duration of rupture of membranes (hours), mean ± SD	5.52 ± 3.19	5.50 ± 3.18	0.961
Post-placental vaginal insertion	132 (88.0%)	132 (88.0%)	
Intra-cesarean insertion	18 (12.0%)	18 (12.0%)	

PBAC scores at follow-up

Six Weeks

At six weeks postpartum, the majority of participants in both groups had not yet resumed menstruation, resulting in PBAC scores of 0 for most women. Consequently, no significant difference in menstrual blood loss was observed between the Cu-T 380A and Cu 375 groups at this early postpartum stage (Table 2). Subgroup analysis based on the route of insertion demonstrated similar findings. Among women who underwent vaginal PPIUCD insertion, PBAC scores remained comparable between the two groups (Table 3). Likewise, no statistically significant difference was observed in the intra-cesarean insertion subgroup (Table 4).

**Table 2:** Pictorial Blood Assessment Chart scores at follow-up visits Data are presented as median (IQR) and range; *p<0.05 is considered statistically significant. Cu-T 380A: Copper-T 380A

Time Point	Cu-T 380A (n=150) Median (IQR), Range	Cu 375 (n=150) Median (IQR), Range	Statistical Test Value	p-value^*^
6 weeks	0 (0–0), 0–62	0 (0–0), 0–40	Wilcoxon Mann-Whitney U Test, W=11654.500	0.18
6 months	50 (43.25–59), 23–79	42.5 (36.25–49.75), 20–67	t-test, T=6.675	<0.001

**Table 3:** Pictorial Blood Assessment Chart scores among women undergoing vaginal postpartum intrauterine contraceptive device insertion Data are presented as median (IQR) and range; *p<0.05 is considered statistically significant. Cu-T 380A: Copper-T 380A

Time Point	Cu-T 380A (n=132) Median (IQR), Range	Cu 375 (n=132) Median (IQR), Range	Statistical Test Value	p-value^*^
6 weeks	0 (0–0), 0–62	0 (0–0), 0–40	Wilcoxon Mann-Whitney U Test, W=8865.500	0.525
6 months	50.5 (44–57.25), 23–79	42 (36–48.25), 20–67	t-test, T=6.832	<0.001

**Table 4:** Pictorial Blood Assessment Chart scores among women undergoing intra-cesarean postpartum intrauterine contraceptive device insertion Data are presented as median (IQR) and range; *p<0.05 is considered statistically significant. Cu-T 380A: Copper-T 380A

Time Point	Cu-T 380A (n=18) Median (IQR), Range	Cu 375 (n=18) Median (IQR), Range	Statistical Test Value	p-value^*^
6 weeks	0 (0–0), 0–55	0 (0–0), 0–0	Wilcoxon Mann-Whitney U Test, W=189.000	0.08
6 months	48 (36.25–62), 28–74	46 (37.75–51), 31–62	t-test, T=1.089	0.285

Six Months

By six months postpartum, menstrual cycles had resumed in most participants. At this time point, women using Cu-T 380A demonstrated significantly higher PBAC scores compared with those using Cu 375, indicating relatively greater menstrual blood loss among Cu-T 380A users (Mann-Whitney U test, p < 0.001) (Table 2). In subgroup analysis, a similar trend was observed among women who underwent vaginal PPIUCD insertion, where Cu-T 380A users reported significantly higher PBAC scores than Cu 375 users (p < 0.001) (Table 3). However, in the intra-cesarean subgroup, PBAC scores were comparable between the two groups, and the difference did not reach statistical significance (p = 0.285) (Table 4).

Dysmenorrhea (VAS) scores

Six Weeks

At six weeks postpartum, dysmenorrhea scores were minimal and comparable between both groups. Since the majority of women had not yet resumed their menses at this follow-up visit, there were no recordable pain scores in most participants. Among those recorded, no statistically significant difference in dysmenorrhea scores was observed between the Cu-T 380A and Cu 375 groups (Table 5). Subgroup analyses based on route of insertion also showed comparable findings in both vaginal and intra-cesarean insertion groups (Tables 6, 7).

**Table 5:** Dysmenorrhea (Visual Analog Scale) scores at follow-up visits Data are presented as median (IQR) and range; *p<0.05 is considered statistically significant. Cu-T 380A: Copper-T 380A

Time Point	Cu-T 380A (n=150) Median (IQR), Range	Cu 375 (n=150) Median (IQR), Range	Statistical Test Value	p-value*
6 weeks	0 (0–0), 0–8	0 (0–0), 0–5	Wilcoxon Mann-Whitney U Test, W=11647.000	0.188
6 months	4 (4–5), 2–8	3 (3–4), 2–5	Wilcoxon Mann-Whitney U Test, W=17071.500	<0.001

**Table 6:** Dysmenorrhea (Visual Analog Scale) scores among women undergoing vaginal postpartum intrauterine contraceptive device insertion Data are presented as median (IQR) and range; *p<0.05 is considered statistically significant. Cu-T 380A: Copper-T 380A

Time Point	Cu-T 380A (n=132) Median (IQR), Range	Cu 375 (n=132) Median (IQR), Range	Statistical Value Test	p-value*
6 weeks	0 (0–0), 0–8	0 (0–0), 0–5	Wilcoxon Mann-Whitney U Test, W=8862.500	0.533
6 months	4 (4–5), 2–8	3 (3–4), 2–5	Wilcoxon Mann-Whitney U Test, W=13377.000	<0.001

**Table 7:** Dysmenorrhea (Visual Analog Scale) scores among women undergoing intra-cesarean postpartum intrauterine contraceptive device insertion Data are presented as median (IQR) and range; *p<0.05 is considered statistically significant. Cu-T 380A: Copper-T 380A

Time Point	Cu-T 380A (n=18) Median (IQR), Range	Cu 375 (n=18) Median (IQR), Range	Statistical Test Value	p-value*
6 weeks	0 (0–0), 0–5	0 (0–0), 0–0	Wilcoxon Mann-Whitney U Test, W=189.000	0.08
6 months	4.5 (4–5), 2–7	3.5 (3–4), 2–5	Wilcoxon Mann-Whitney U Test, W=225.500	0.04

Six Months

However, at six months postpartum follow-up, dysmenorrhea scores were significantly higher among Cu-T 380A users compared with Cu 375 users (Mann-Whitney U test, p < 0.001), suggesting greater menstrual pain associated with Cu-T 380A (Table 5). Subgroup analysis showed that this difference was statistically significant irrespective of the route of PPIUCD insertion, where Cu-T 380A users reported higher dysmenorrhea scores compared with Cu 375 users (p < 0.001) in vaginal PPIUCD (Table 6) as well as in the intra-cesarean PPIUCD subgroup (p = 0.040) (Table 7).

Secondary outcomes

The overall occurrence of adverse outcomes was low in both groups, and no statistically significant differences were observed between Cu-T 380A and Cu 375 users at both follow-up visits (Table 8). Although abnormal vaginal discharge was reported by a slightly higher proportion of participants in the Cu-T 380A group as compared to Cu 375, the findings were statistically nonsignificant. Rates of IUCD expulsion were low and comparable between groups at both follow-up visits. Whereas there were no requests for IUCD removal at the six-week follow-up, a higher percentage of Cu-T 380A users requested IUCD removal at the six-month follow-up visit. However, this difference did not reach statistical significance (p>0.05). The main reason cited for removal was heavy menstrual blood loss in both groups. There were no cases of uterine perforation or unintended pregnancy in either group during the study period.

**Table 8:** Secondary outcomes following postpartum intrauterine contraceptive device insertion # Fisher’s exact test (X2) was applied; Data are presented as number and percentage; *p<0.05 is considered statistically significant. Cu-T 380A: Copper-T 380A

Outcome	6 Weeks Cu-T 380A, n (%)	6 Weeks Cu 375, n (%)	p-value	6 Months Cu-T 380A, n (%)	6 Months Cu 375, n (%)	p-value*
Abnormal vaginal discharge	5 (3.8%)	2 (1.5%)	0.448^#^	7 (4.7%)	5 (3.3%)	0.556^#^
Intrauterine contraceptive device expulsion	1 (0.7%)	0 (0.0)	1.000^#^	1 (0.7%)	3 (2.0%)	0.622^#^
Unintended pregnancy	0	0	-	0	0	-
Uterine perforation	0	0	-	0	0	-
Request for removal (6 months only)	-	-	-	5 (3.3%)	3 (2.0%)	0.723^#^

Satisfaction scores

Patient satisfaction with the PPIUCD remained high in both groups (median score of 4 with scores recorded as 0-5). Satisfaction scores were statistically comparable between Cu-T 380A and Cu 375 users at both six-week and six-month follow-up visits (Table 9). When asked regarding the likelihood of recommending PPIUCD as a method of choice for postpartum contraception to other women, recommendation rates were overwhelmingly positive in both Cu-T 380A (96%) and Cu 375 users (98%). These findings suggest that both devices were very well accepted by postpartum women.

**Table 9:** Patient satisfaction scores (0-5) at follow up visits Data are presented as median and interquartile range; *p<0.05 is considered statistically significant. Cu-T 380A: Copper-T 380A

Time Point	Cu-T 380A (n=150) Median (IQR), Range	Cu 375 (n=150) Median (IQR), Range	Wilcoxon Mann-Whitney U Test	P-value*
6 weeks	4(4-4), 3-5	4(4-4), 3-5	W=10926.500	0.563
6 months	4(4-4), 2-5	4(4-4),2-5	W=10802.500	0.449

## Discussion

In this prospective comparative study of 300 postpartum women undergoing immediate PPIUCD insertion, both Cu-T 380A and Cu 375 demonstrated high safety, contraceptive efficacy, and patient satisfaction over six months of follow-up. While short-term outcomes at six weeks were comparable, statistically significant differences in menstrual blood loss and dysmenorrhea emerged at six months, with higher PBAC and VAS scores observed among Cu-T 380A users.

Our findings are broadly consistent with interval insertion studies demonstrating higher bleeding and dysmenorrhea scores with Cu-T 380A compared with Cu 375. In a randomized clinical trial by Shahnazi et al., where a comparison of bleeding and dysmenorrhea with Cu T 380A and Cu 375 was done in 48 women seeking IUCD as a contraceptive, results found a significantly lower score of bleeding and reduced severity and duration of dysmenorrhea with Cu 375 than Cu-T 380A [8]. Similar observations were noted by Elsherbieny et al. in 110 participants, where Cu T 380A was associated with significantly increased duration and amount of bleeding as compared to Cu 375 IUCD (P < 0.001) [9]. However, evidence in the immediate postpartum setting remains heterogeneous, as only a limited number of studies have directly compared the effects of different copper IUDs in the immediate postpartum period [10]. Some postpartum studies have reported comparable bleeding patterns between devices, whereas others have suggested higher early bleeding with Cu-T 380A. In a multicentric trial conducted by the UK Family Planning and Reproductive Health Research Network, insertion of PPIUCDs demonstrated that Cu-T 380A was associated with higher bleeding intensity compared with Cu 375 [11]. Conversely, El Beltagy et al. evaluated 300 women randomized to receive either Cu-T 380A or Cu 375 within 48 hours postpartum and reported no significant differences in overall bleeding patterns between the groups at both six-week and six-month follow-up. However, excessive bleeding was noted more frequently among Cu-T 380A users at the six-week interval [12]. Variability in assessment tools, follow-up duration, and study populations may explain these inconsistencies. The use of a standardized PBAC tool in the present study strengthens comparability and provides quantitative bleeding assessment.

The difference in PBAC scores was statistically significant; however, the absolute magnitude of the difference (approximately 8 points) was relatively small and did not reach thresholds typically considered clinically meaningful. Moreover, median PBAC values in both groups remained well below the conventional cutoff of 100 associated with heavy menstrual bleeding. These findings suggest differences in bleeding intensity between the two PPIUCDs rather than clinically significant menorrhagia.

The greater bleeding and dysmenorrhea observed with Cu-T 380A may be biologically plausible given its higher copper surface area and potential for enhanced local endometrial inflammatory response. Increased prostaglandin-mediated vascular changes and uterine contractility may contribute to both bleeding intensity and pain perception. Structural differences between devices, including variations in copper surface area and frame flexibility, may influence endometrial contact and inflammatory response during postpartum involution [13].

Subgroup analysis revealed that differences in bleeding and dysmenorrhea were more pronounced following vaginal insertions, whereas intra-cesarean insertions showed smaller between-group variation. This was similar to the results of a comparative study by Al-Hussaini comparing 143 women for intra-cesarean section insertion of Cu-T 380 versus Cu 375 IUD, which found no difference between the two groups at six months' follow-up in menstrual patterns [14]. These observations may reflect differences in uterine remodeling and healing dynamics following cesarean delivery; however, this hypothesis requires further investigation.

Importantly, expulsion, infection-related discharge, removal rates, and unintended pregnancy were low and comparable between groups. These findings were consistent with the results of the study by El Belgaty et al., where no significant difference was observed between the IUDs regarding safety, efficacy, expulsion, bleeding, or perforation [12]. Similarly, Divya et al. found no significant difference between Cu 375 and Cu-T 380A regarding safety, efficacy, and complications in a prospective, randomized comparative study of post-placental Cu-T 380A and Cu 375 IUCD in 300 primiparous women delivering by cesarean section who were followed up at one month, three months, six months, and 12 months [15]. 

Patient satisfaction remained high in both arms, suggesting that both devices are acceptable postpartum contraceptive options.

In summary, both Cu-T 380A and Cu 375 are safe and effective options for immediate postpartum contraception. While Cu 375 was associated with modestly lower bleeding and dysmenorrhea scores at six months, these differences did not reflect clinically significant menorrhagia. The findings are best interpreted as differences in tolerability rather than safety or efficacy. Incorporating device-specific bleeding profiles into postpartum counseling may facilitate shared decision-making and individualized contraceptive selection.

Strengths

Strengths of this study include its prospective design, relatively large sample size, and quantitative bleeding assessment using PBAC. The inclusion of subgroup analysis by route of insertion further enhances clinical interpretability.

Limitations

Breastfeeding status, which may influence postpartum menstrual resumption and bleeding patterns, was not quantitatively adjusted for and may represent a confounding factor. Additionally, follow-up was limited to six months, and longer-term continuation outcomes need to be assessed. The study was limited to a single center; hence, a multicentric study will help in improving the analysis of the outcome in different regional population groups.

## Conclusions

Although both the PPIUCD options CuT-380A and Cu 375 have good patient acceptability and high satisfaction scores, Cu 375 as PPIUCD causes a significantly lower menstrual blood loss, as well as severity and duration of dysmenorrhea, when compared to Cu-T 380A. Since complications at follow-up are a major determinant in PPIUCD acceptance, Cu 375 is a lucrative option for women opting for reversible contraception after delivery.
